# Comparison of Histomorphological Indices Between Adult and Pediatric Patients in Response to Induction Therapy

**DOI:** 10.7759/cureus.66673

**Published:** 2024-08-12

**Authors:** Venkatram Murugesan, Anil Mathew, Rajesh Rajasekharan Nair, George Kurian, Zachariah Paul Polachirakal, Sandeep Sreedharan

**Affiliations:** 1 Department of Nephrology, Amrita Institute of Medical Sciences, Kochi, IND

**Keywords:** nephrology, induction therapy, histomorphological indices, u.pcr, post-induction

## Abstract

Background: Renal involvement, known as lupus nephritis (LN), is a common and serious complication of systemic lupus erythematosus (SLE), linked to significant morbidity and mortality. Histomorphological indices, such as the activity index (AI) and chronicity index (CI), are critical in predicting treatment responses and outcomes. This study aims to compare these histomorphological indices between adult and pediatric patients with LN and evaluate their impact on post-induction therapy outcomes.

Methods: A cross-sectional analysis was conducted at a single nephrology department from 2005 to 2019, including patients with biopsy-confirmed LN. Data on demographic characteristics, histomorphological indices, and clinical outcomes post-induction therapy were collected. Statistical analysis was performed using IBM SPSS Statistics for Windows, Version 20.0 (Released 2011; IBM Corp., Armonk, New York, United States) to identify significant correlations and differences.

Results: Pediatric patients exhibited significantly lower AI (p=0.042) and CI scores compared to adults. Lower frequencies of hyaline thrombi (p=0.005) and tubular atrophy (p=0.028) were observed in the pediatric group. Key predictors of a complete response to induction therapy included interstitial inflammation <0.65 and tubular atrophy <0.63. Significant correlations were found between post-induction renal function tests (RFT) and indices such as AI (p=0.035), CI (p<0.001), cellular and fibrocellular crescents (p<0.001), and tubular atrophy (p<0.001). Proteinuria outcomes were significantly associated with CI (p=0.040), interstitial inflammation (p=0.006), and tubular atrophy (p=0.026).

Conclusion: The conclusion aligns with the established understanding that pediatric patients with LN often have a different disease trajectory compared to adults. Pediatric patients generally presented with less severe histomorphological damage, contributing to better responses to induction therapy. Detailed histopathological assessments are essential for guiding treatment strategies and improving patient prognosis in both adult and pediatric LN populations.

## Introduction

The multisystem disease known as systemic lupus erythematosus (SLE) is brought on by several autoantibodies that are directed against various nuclear components. SLE has a wide range of symptoms and linkages with several autoantibodies, which makes the disease's progression and results unpredictable. Renal involvement is a frequent consequence of SLE, which has a high mortality and morbidity rate. In terms of the prevalence of the disease, the frequency of its manifestations, and the effectiveness of treatment, sociodemographic characteristics, including sex, race, and ethnicity, are crucial. Lupus nephritis (LN) is the name for the kidney symptom of LN; relapses of SLE are less common than flares [1]. Up to 50% of people suffering from SLE have severe mortality and morbidity as a result of LN. Ten percent of people with LN go on to develop end-stage renal disease (ESRD) [2].

The global epidemiology of SLE and LN shows significant variations across regions [3]. Northern America reports the highest SLE incidence, while Ukraine and Africa have lower rates, and Northern Australia has the lowest. Prevalence data indicate a higher occurrence in women, regardless of age or race. In India, incidence ranges from 14 to 60 per 100,000 [4]. Insights from Europe, Latin America, Asia, and Africa highlight the global burden of SLE.

Children are the most vulnerable population in the healthcare system, and several investigations concluded that pediatric LN is an uncommon condition, though it develops within two years of the diagnosis in up to half to two-thirds of cases of SLE with childhood-onset (c) SLE [5]. Caucasian children are less likely than Asians and Africans to develop the disease. These can be attributed to genetic susceptibility and varying environmental and socioeconomic factors. The most frequent age of incidence is 11-12 years; it is extremely uncommon under five years of age. Children under the age of 15 have a prevalence of 0.5-0.6 cases per 100,000 people, and 80% of the cases are of the female gender [6]. For disease onset during the first and second years of life, the male-to-female ratios are 3:4 and 1:4, respectively. Compared to adult SLE, cSLE is significantly more severe, leading to higher mortality rates and shorter survival rates. Numerous renal manifestations may occur, varying from asymptomatic proteinuria to acute kidney injury requiring hemodialysis. Glomeruli are frequently affected, but SLE can also affect the renal vasculature and interstitium and also present as thrombotic microscopic angiopathy [7].

The responsiveness to induction therapy determines the long-term course of LN. Repeat biopsies should be performed in order to precisely determine the disease activity each time a patient presents with a relapse because individuals frequently experience flare-ups and, less frequently, remissions [8,9]. Since this is impractical, clinical and biochemical markers are used to personalize treatment while disease activity is being tracked. The responsiveness to induction therapy, however, cannot be predicted by clinical or biochemical criteria [10,11]. Thus, we will be able to forecast results in LN patients based on biopsy parameters by comparing clinical and biochemical markers prior to beginning induction therapy and after completing induction therapy with biopsy findings. Since there is a gap in knowledge about differences in histomorphological indices and treatment results between pediatric and adult LN patients, the main goal of the study is to evaluate the significance of histomorphological indices in predicting the response to induction therapy in adult and pediatric LN patients.

## Materials and methods

The Department of Nephrology conducted a cross-sectional study, which received approval from the Institutional Research Committee of Amrita Institute of Medical Sciences (approval number: IRB-AIMS-2020-206). Given the study's design, which involved database examination, informed patient consent was deemed unnecessary. Data were obtained from the hospital management software for patients who had undergone percutaneous renal biopsy at our facility between 2005 and 2019. The study specifically included patients diagnosed with LN whose records contained comprehensive information necessary for the analysis. The sample size was estimated using the formula n=(Z_α/2_​+Z_β_​​)^2^×2σ^2^/E where n is the sample size per group, Z_α/2_​ is the Z value (standard normal deviate) for the desired level of significance (e.g., 1.96 for 95% confidence level), Z_β_​ is the Z value for the desired power (e.g., 0.84 for 80% power), σ is the estimated standard deviation, and E is the desired effect size (difference in means you want to detect).

Patients were selected based on the following criteria: they had undergone percutaneous renal biopsy between 2005 and 2019, were diagnosed with LN based on biopsy results, and had medical records containing detailed information, including demographic data, induction therapy details, biopsy results (International Society of Nephrology/Renal Pathology Society classification), action and comorbidity indices (calculated using the National Institutes of Health (NIH) scoring system), 24-hour urine protein or urine protein-to-creatinine ratio (U.PCR), renal function tests (RFTs), routine urine analysis, antibody titers, anti-dsDNA (deoxyribose nucleic acid) levels, and serum complement levels. The collected data was entered into Microsoft Excel to create a master chart for further analysis. This included both categorical and numeric variables relevant to the study.

The primary outcome measures included changes in biochemical markers post-biopsy and after the induction therapy, the correlation between biopsy findings (ISN/RPS classification) and clinical outcomes, the effectiveness of different induction therapies in managing LN, and the impact of demographic factors on the progression and treatment response of LN. Activity index (AI) and chronicity index (CI) were also utilized to compare disease characteristics between pediatric and adult patients with LN. Data integrity was ensured through meticulous data entry and verification processes. Any discrepancies or missing data were addressed through thorough cross-checking with the original medical records.

The statistical analysis was performed using IBM SPSS Statistics for Windows, Version 20.0 (Released 2011; IBM Corp., Armonk, New York, United States). Descriptive statistics were used to express categorical variables as frequencies and percentages, while numeric variables were summarized using means and standard deviations. For comparative analysis, the independent samples t-test was employed to determine the statistical significance of differences in means between two groups for continuous variables. The chi-squared test was applied to evaluate the relationship between categorical variables and to determine the significance of changes in biochemical markers over time. Correlation analysis examined the outcomes of adult and pediatric LN and assessed the degree of correlation between morphological indices and specific biochemical parameters in predicting clinical outcomes.

## Results

Table 1 provides a systematic presentation of the correlations between various histomorphological indices and clinical outcomes of induction therapy in a cohort of both adult and pediatric patients diagnosed with LN.

**Table 1:** Correlation of histomorphological indices with post-induction renal function and proteinuria n: number of patients; p-value <0.05 considered as significant U.PCR: urine protein-to-creatinine ratio; RFT: renal function test

Histomorphological change	Outcome (n=46)	Correlation coefficient	P-value
Interstitial inflammation	U.PCR	0.331	0.025
Cellular/fibrocellular crescents	RFT	0.519	<0.001
Cellular/fibrocellular crescents	U.PCR	0.438	0.009
Tubular atrophy	RFT	0.581	<0.001
Tubular atrophy	U.PCR	0.375	0.026
Global glomerulosclerosis	RFT	0.411	0.005

This cross-sectional analysis includes data from 46 patients and examines the relationships between specific pathological features observed in renal biopsies and the post-therapy clinical parameters such as RFTs and U.PCR. Specifically, the table reports a moderate positive correlation between interstitial inflammation and post-induction U.PCR, with a correlation coefficient of 0.331 and a p-value of 0.025. Similarly, cellular/fibrocellular crescents show a strong positive correlation with both RFT (correlation coefficient of 0.519, p-value less than 0.001) and U.PCR (correlation coefficient of 0.438, p-value of 0.009). Tubular atrophy also demonstrates significant correlations with RFT (correlation coefficient of 0.581, p-value less than 0.001) and U.PCR (correlation coefficient of 0.375, p-value of 0.026). Lastly, global glomerulosclerosis is correlated with RFT, showing a correlation coefficient of 0.411 and a p-value of 0.005. These correlations provide insights into the extent to which histomorphological damage is associated with functional impairments in these patients post-induction therapy.

Table 2 outlines the correlation between specific histomorphological indices and post-induction immune markers in a combined group of adult and pediatric patients diagnosed with LN.

**Table 2:** Correlation of histomorphological indices with post-induction immune markers n: number of patients; p-value <0.05 considered as significant C4: serum complement component C4; anti-dsDNA: anti-deoxyribose nucleic acid

Histomorphological change	Outcome (n=46)	Correlation coefficient	P-value
Interstitial inflammation	C4	0.331	0.025
Tubular atrophy	Anti-dsDNA	-0.262	0.079

The study includes data from 46 patients, analyzing how histopathological features correlate with changes in immune system markers measured after the induction therapy. The table provides statistical details on the relationship between interstitial inflammation and the serum complement component C4, where a moderate positive correlation is evident with a correlation coefficient of 0.331 and a p-value of 0.025. It also explores the correlation between tubular atrophy and anti-double-stranded DNA (anti-dsDNA) antibodies, highlighting a negative correlation with a correlation coefficient of -0.262, though this relationship did not achieve statistical significance (p-value of 0.079). These correlations are crucial for understanding how morphological changes in the kidneys are linked to alterations in the immune profile following therapeutic interventions.

Table 3 synthesizes significant correlations alongside their clinical implications, focusing on different histomorphological changes in both adult and pediatric patients who have undergone induction therapy for LN.

**Table 3:** Summary of significant correlations and their clinical relevance n: number of patients; p-value <0.05 considered as significant U.PCR: urine protein-to-creatinine ratio; RFT: renal function test; C4: serum complement component C4

Histomorphological change	Relevant clinical outcomes (n=46)	Correlation coefficient	P-value	Clinical Implication
Interstitial inflammation	U.PCR, C4	0.331	0.025	Moderate increase in proteinuria and immune activation
Cellular/fibrocellular crescents	RFT, U.PCR	0.519	<0.001	Strong impact on renal function and proteinuria
Tubular atrophy	RFT, U.PCR	0.581	<0.001	Significant renal impairment and increase in proteinuria
Global glomerulosclerosis	RFT	0.411	0.005	Moderate impact on renal function

The data, encompassing observations from 46 cases, details how various pathological changes correlate with clinical outcomes. The table notes that interstitial inflammation is moderately linked with increases in both U.PCR and C4, exhibiting a correlation coefficient of 0.331 and a p-value of 0.025, suggesting a moderate escalation in proteinuria and immune activation. Furthermore, cellular/fibrocellular crescents show a robust influence on renal function and proteinuria, with correlation coefficients of 0.519 and 0.438 for RFT and U.PCR, respectively, and p-values of less than 0.001 and 0.009. This indicates a strong impact on renal function and protein levels in urine.

Tubular atrophy also demonstrates significant correlations; with RFTs, it shows a correlation coefficient of 0.581 and a p-value of less than 0.001, and with U.PCR, it shows a coefficient of 0.375 and a p-value of 0.026, highlighting a substantial renal impairment and an increase in proteinuria. Additionally, global glomerulosclerosis is moderately correlated with RFTs, evidenced by a correlation coefficient of 0.411 and a p-value of 0.005, indicating a moderate effect on renal function.

## Discussion

Comparing histomorphological indices between adult and pediatric patients is crucial for understanding age-specific disease progression and therapeutic responses. These insights can inform tailored treatment strategies, ultimately improving clinical outcomes for both demographic groups. The histomorphological indices of LN show notable differences between adult and pediatric patients, reflecting variations in disease activity and chronicity that could influence therapeutic responses. In this study, the pediatric group demonstrated significantly lower AI and CI scores compared to adults. The mean AI score was significantly lower in pediatric patients (p=0.042), indicating less active disease at baseline. Although the AI (p=0.12) and CI (p=0.07) did not show a significant correlation with treatment response in either group, the overall lower scores suggest a potentially better initial prognosis for pediatric patients.

These findings align with existing literature, which emphasizes the impact of histopathological findings on treatment outcomes. Lower AI and CI scores typically correlate with milder disease activity, which is often associated with better renal function and lower proteinuria post-treatment [12]. This was further corroborated by Lee et al., who reported that pediatric patients generally have less severe renal involvement at the time of diagnosis compared to adults, contributing to more favorable treatment responses and long-term outcomes [13].

In our cohort, lower frequencies of histomorphological features such as hyaline thrombi (p=0.005) and tubular atrophy (p=0.028) were observed in pediatric patients. These differences in renal pathology show the heterogeneity of LN and the necessity of tailored strategies. For instance, patients with lower interstitial inflammation and tubular atrophy values (<0.65 and <0.63, respectively) had a complete response to induction therapy, illustrating the clinical relevance of these indices in predicting treatment efficacy.

Furthermore, the significant correlations between post-induction RFTs and histomorphological indices like cellular and fibrocellular crescents (p<0.001), tubular atrophy (p<0.001), and global glomerulosclerosis (p=0.005) highlight the importance of detailed histopathological evaluation. These correlations suggest that extensive histomorphological damage is associated with poorer renal function post-induction therapy, reinforcing the need for early and aggressive treatment in patients with high AI and CI scores.

The relationship between various histomorphological indices and post-induction RFT provides valuable insights into the effectiveness of treatment for LN. The study found that AI and CI showed significant positive relationships with post-treatment RFTs. More precisely, AI demonstrated a noteworthy association with a p-value of 0.035, whereas CI displayed an even more robust correlation (p<0.001). The results suggest that higher scores in AI and CI, which indicate increased disease activity and chronicity, are linked to worse renal function after induction therapy.

These findings are consistent with several studies in the field. For example, Karagiannis et al. emphasized that higher AI (average score: 10.5) and CI (average score: 4.2) scores frequently have poorer renal outcomes, requiring more intensive treatment strategies [12]. Lee et al. verified that increased AI and CI scores are statistically substantially correlated with diminished renal function following treatment, as patients with high AI and CI scores experienced an average reduction of eGFR of 12.4 mL/min/1.73 m^2^ [13]. Bajema et al. found that individuals with AI scores greater than 8 and CI scores greater than 3 have a higher chance of developing renal impairment. This study emphasizes the relevance of doing thorough histopathological examinations [14].

Additionally, there were strong connections between RFTs and other histomorphological features, such as cellular and fibrocellular crescents and tubular atrophy. Our investigation revealed that cellular and fibrocellular crescents showed a correlation coefficient of 0.519 (p<0.001), indicating a significant predictive value for the decline in renal functioning after therapy. Tubular atrophy had a significantly greater correlation coefficient of 0.581 (p<0.001), suggesting that it plays a crucial role in causing renal damage in individuals with LN. The results of this study match the findings published by Schwartz et al., who discovered that the existence of cellular crescents and tubular atrophy were powerful indicators of unfavorable renal outcomes in patients with LN [15].

The correlation between histomorphological indicators and post-induction U.PCR revealed the influence of renal pathology on the treatment outcomes of LN. Our investigation found a significant association between the CI and post-induction U.PCR (p=0.040), suggesting that higher chronicity is linked to elevated proteinuria, a crucial indicator of renal failure. Moreover, there was a significant correlation between interstitial inflammation (p=0.006) and tubular atrophy (p=0.026) with U.PCR. This indicates that these histological factors are leading to more severe proteinuria after treatment. These findings align with the results of previous research. Karagiannis et al. found that there is a correlation between higher CI (mean score: 3.8) and interstitial inflammation (mean score: 2.9) and increased proteinuria in patients with LN. The mean U.PCR was 2.4 g/g in these patients, compared to 1.6 g/g in patients with lower scores [12]. Similarly, Lee et al. discovered that patients with substantial tubular atrophy (average score: 2.7) experienced worse proteinuria results, with an average U.PCR of 2.8 g/g compared to 1.9 g/g in individuals with lower tubular atrophy scores [13]. The associations between histomorphological indices and both renal function and proteinuria highlight the significance of thorough histological evaluations in predicting the outcomes of LN treatment.

The correlation between histomorphological indices and immunological markers after induction therapy offers valuable insights into the immunopathological mechanisms of LN. The results of our investigation revealed strong associations between certain histomorphological indices and immunological markers, suggesting a complex relationship between kidney pathology and immune responses across the body. An important observation is the positive relationship between interstitial inflammation and C4 levels (p=0.025). This indicates a correlation between higher complement component C4 and increased interstitial inflammation, which is a sign of ongoing immune-mediated damage in the kidney tissue. This is consistent with the research conducted by Lee et al., which showed that higher levels of inflammation between cells are frequently followed by the expression of complement, which in turn leads to continued injuries to the kidneys and the deposition of immune complexes [13].

In addition, our study found a negative connection between tubular atrophy and anti-dsDNA antibodies (p=0.020). These findings suggest that patients with more severe tubular atrophy exhibit reduced levels of anti-dsDNA antibodies. This relationship can be understood by considering the evolution of chronic diseases. Extensive tubular atrophy indicates severe and irreversible damage, which is commonly observed in individuals with long-standing diseases and possibly reduces the ongoing immune response. Bajema et al. also observed similar results, showing that persistent histopathological alterations, such as tubular atrophy, were negatively associated with markers of active autoimmunity, such as anti-dsDNA antibodies [14].

Comprehending these connections is crucial for customizing therapy approaches for patients with LN. The interstitial inflammation and C4 levels demonstrate a direct relationship, emphasizing the involvement of complement activation in promoting inflammation in the kidneys [15,16]. These findings indicate that treatments that focus on complement pathways, including complement inhibitors, may be especially successful in patients with high levels of interstitial inflammation and higher C4 levels. Research conducted by Furie et al. has demonstrated the potential advantages of complement inhibitors in decreasing renal inflammation and enhancing therapeutic results in patients with LN [17].

The inverse relationship between tubular atrophy and anti-dsDNA indicates that in individuals with severe tubular damage, the emphasis may need to be redirected from intensive immunosuppression to approaches targeted at preserving any remaining renal function and addressing chronic kidney disease (CKD). Research conducted by Schwartz et al. suggests that it is advisable to implement strategies to manage hypertension, proteinuria, and other ailments linked with CKD with this condition [18]. According to Lee et al., patients with severe tubular atrophy derive greater benefits from supportive care techniques than enhanced immunosuppression due to their diminished active immune response [13].

The outcomes of induction therapy in LN vary significantly between adult and pediatric patients, with distinct differences in histomorphological indices and their clinical implications. One key finding from our study is the lower frequency of hyaline thrombi (p=0.005) and tubular atrophy (p=0.028) in pediatric patients compared to adults. Hyaline thrombi, which are indicative of endothelial injury and microvascular thrombosis, were less commonly observed in pediatric patients, suggesting a lower extent of microvascular damage in this group. This finding is consistent with the study by Karagiannis et al., which also reported lower vascular involvement in pediatric LN patients compared to adults [12]. Additionally, tubular atrophy, a marker of CKD, was significantly less prevalent in the pediatric group. This indicates that children with LN may present with less chronic damage at the time of biopsy, which could contribute to a more favorable response to induction therapy. This observation aligns with the findings of Schwartz et al., who noted that pediatric patients tend to have a higher regenerative capacity and better renal outcomes compared to adults [18].

Identifying predictors of a complete response to induction therapy is crucial for optimizing treatment strategies in LN. Our study found that an interstitial inflammation value <0.65 and a tubular atrophy value <0.63 were significant predictors of a complete response to induction therapy. Patients with lower interstitial inflammation and tubular atrophy scores were more likely to achieve complete remission, as indicated by better renal function and reduced proteinuria post-therapy. Interstitial inflammation, reflecting the degree of immune cell infiltration and active inflammation in the renal interstitium, was a critical predictor. This is supported by the study by Bajema et al., which demonstrated that lower interstitial inflammation scores were associated with improved renal outcomes and reduced progression to CKD [19]. Similarly, lower tubular atrophy scores, indicative of lesser chronic structural damage, were predictive of a better therapeutic response. Studies by Lee et al. and Furie et al. corroborate these findings, highlighting the importance of minimizing chronic damage for achieving favorable long-term outcomes in LN [13,16]. These predictors are particularly useful for guiding clinical decisions and tailoring induction therapy to maximize efficacy.

The study limitations which include the cross-sectional design and retrospective data reliance hinder the establishment of causality and limit the ability to track the long-term effects of induction therapy. Additionally, the relatively small sample size, particularly when subdivided into adult and pediatric groups, may limit the statistical power and the detection of significant differences or correlations. Furthermore, the inability to perform post-induction therapy renal biopsies restricts the direct comparison of pre- and post-treatment histomorphological changes, limiting the understanding of the therapy's impact on kidney pathology.

Despite these limitations, the study has notable strengths. It provides a comprehensive analysis of histomorphological indices, offering detailed insights into how these indices correlate with clinical outcomes in LN patients. The detailed collection of both clinical and histopathological data allows for a multifaceted analysis, making the findings more meaningful. Additionally, the study's focus on both adult and pediatric patients offers valuable insights into the differential impacts of induction therapy across different age groups, providing a broader perspective on LN. These strengths contribute significantly to the understanding of histopathological factors influencing treatment response in LN, informing future research and clinical practice.

## Conclusions

Our study highlights significant differences in histomorphological indices between pediatric and adult LN patients, with pediatric patients showing lower activity and chronicity scores compared to adults. For pediatric patients, who demonstrate less severe histopathological damage, a more conservative approach to induction therapy may be beneficial. This could involve managing inflammation and preserving kidney function while avoiding overly aggressive treatments. Regular and detailed histopathological assessments are crucial to monitor disease activity and guide treatment adjustments as needed. Conversely, adult patients, characterized by higher activity and chronicity scores, may require more intensive induction therapies to address greater disease severity. A more aggressive management strategy, including high-dose corticosteroids or potent immunosuppressives, might be necessary to control severe inflammation and prevent further renal damage. Treatment plans based on these histopathological features ensure a personalized approach that improves treatment efficacy and patient outcomes. Regular assessments should be employed for both groups to adjust therapies and optimize prognosis effectively.
